# Multicenter, Observational Cohort Study Evaluating Third-Generation Cephalosporin Therapy for Bloodstream Infections Secondary to *Enterobacter*, *Serratia*, and *Citrobacter* Species

**DOI:** 10.3390/antibiotics9050254

**Published:** 2020-05-14

**Authors:** Caroline Derrick, P. Brandon Bookstaver, Zhiqiang K. Lu, Christopher M. Bland, S. Travis King, Kayla R. Stover, Kathey Rumley, Shawn H. MacVane, Jenna Swindler, Scott Kincaid, Trisha Branan, David Cluck, Benjamin Britt, Kelly E. Pillinger, Bruce M. Jones, Virginia Fleming, V. Paul DiMondi, Sandy Estrada, Brad Crane, Brian Odle, Majdi N. Al-Hasan, Julie Ann Justo

**Affiliations:** 1 Department of Medicine, University of South Carolina School of Medicine Columbia, SC 29203, USA; caroline.derrick@uscmed.sc.edu (C.D.); majdi.alhasan@uscmed.sc.edu (M.N.A.-H.); 2 Department of Clinical Pharmacy and Outcomes Sciences, University of South Carolina College of Pharmacy, Columbia, SC 29208, USA; bookstaver@cop.sc.edu (P.B.B.); lu32@email.sc.edu (Z.K.L.); 3 Prisma Health Richland, Columbia, SC 29203, USA; 4 Department of Clinical and Administrative Pharmacy, University of Georgia College of Pharmacy, Savannah, GA 31324, USA; cmbland@uga.edu; 5 St. Joseph’s/Candler Health System, Savannah, GA 31405, USA; jonesbru@sjchs.org; 6 Department of Pharmacy Practice, University of Mississippi School of Pharmacy, Jackson, MS 39216, USA; samuel.king@ochsner.org (S.T.K.); kstover@umc.edu (K.R.S.); 7 Vidant Medical Center, Greenville, NC 27835, USA; kfulton@vidanthealth.com; 8 Department of Pharmacy Practice, Campbell University College of Pharmacy and Health Sciences, Buies Creek, NC 27506, USA; VDimondi@ITS.JNJ.com; 9 Department of Pharmacy, Medical University of South Carolina, Charleston, SC 29425, USA; shawn.macvane@gmail.com; 10 McLeod Regional Medical Center, Florence, SC 29506, USA; jenswindler@mcleodhealth.org; 11 University of Kentucky Healthcare, Lexington, KY 40536, USA; seki232@uky.edu; 12 College of Pharmacy, University of Georgia, Athens, GA 30602, USA; tbranan@uga.edu (T.B.); vfleming@rx.uga.edu (V.F.); 13 Department of Pharmacy Practice, Bill Gatton College of Pharmacy, East Tennessee State University, Johnson City, TN 37614, USA; cluckd@mail.etsu.edu (D.C.); ODLE@mail.etsu.edu (B.O.); 14 Lexington Medical Center, West Columbia, SC 29169, USA; bbbritt@lexhealth.org; 15 Carolinas HealthCare System, Charlotte, NC 28203, USA; kelly_pillinger@urmc.rochester.edu; 16 WakeMed Health and Hospitals, Raleigh, NC 27610, USA; 17 Lee Health, Fort Myers, FL 33901, USA; sestrada@t2biosystems.com; 18 Blount Memorial Hospital, Maryville, TN 37804, USA; crane2001@gmail.com

**Keywords:** bacteremia, cephalosporins, sepsis, beta-lactamases, AmpC, carbapenems

## Abstract

**Objectives:** There is debate on whether the use of third-generation cephalosporins (3GC) increases the risk of clinical failure in bloodstream infections (BSIs) caused by chromosomally-mediated AmpC-producing Enterobacterales (CAE). This study evaluates the impact of definitive 3GC therapy versus other antibiotics on clinical outcomes in BSIs due to *Enterobacter*, *Serratia*, or *Citrobacter* species. **Methods:** This multicenter, retrospective cohort study evaluated adult hospitalized patients with BSIs secondary to *Enterobacter*, *Serratia*, or *Citrobacter* species from 1 January 2006 to 1 September 2014. Definitive 3GC therapy was compared to definitive therapy with other non-3GC antibiotics. Multivariable Cox proportional hazards regression evaluated the impact of definitive 3GC on overall treatment failure (OTF) as a composite of in-hospital mortality, 30-day hospital readmission, or 90-day reinfection. **Results:** A total of 381 patients from 18 institutions in the southeastern United States were enrolled. Common sources of BSIs were the urinary tract and central venous catheters (78 (20.5%) patients each). Definitive 3GC therapy was utilized in 65 (17.1%) patients. OTF occurred in 22/65 patients (33.9%) in the definitive 3GC group vs. 94/316 (29.8%) in the non-3GC group (*p* = 0.51). Individual components of OTF were comparable between groups. Risk of OTF was comparable with definitive 3GC therapy vs. definitive non-3GC therapy (aHR 0.93, 95% CI 0.51–1.72) in multivariable Cox proportional hazards regression analysis. **Conclusions:** These outcomes suggest definitive 3GC therapy does not significantly alter the risk of poor clinical outcomes in the treatment of BSIs secondary to *Enterobacter, Serratia*, or *Citrobacter* species compared to other antimicrobial agents.

## 1. Introduction

Antimicrobial resistance is an increasingly prevalent problem and a substantial contributor to the global disease burden and patient mortality [1]. In particular, beta-lactamase-mediated resistance has emerged as a major threat to patient care. These enzymes may be produced from chromosomal genes or from mobile genetic elements (i.e., transposons, plasmids). Among the chromosomally-mediated beta-lactamases, the AmpC family represents one of the most commonly encountered resistance enzymes [2]. These beta-lactamases are characteristically found in *Enterobacter, Serratia,* or *Citrobacter* spp. [2,3]. Expression of these enzymes is a complex process regulated by transcription factors (AmpR) and regulatory enzymes (AmpD), which work to promote or reduce enzyme production, respectively. Induction of these enzymes may occur following exposure to certain beta-lactams. Additionally, mutations in AmpD, and less commonly AmpR, lead to overexpression of these enzymes, conferring resistance to most beta-lactams, including third-generation cephalosporins (3GC) [2,3,4]. Clinical laboratories do not have a standard detection method for AmpC overproduction among Enterobacterales, leading to a potential underestimation of both the prevalence and degree of expression of this resistance mechanism. Prior data suggest the on-therapy emergence of 3GC resistance can occur despite initial in vitro susceptibility. Complicating this controversy is a relative dearth of clinical evidence to guide the treatment of non-*Enterobacter* AmpC-producing organisms [5,6,7,8,9]. These data highlight the continued controversy surrounding the optimal antimicrobial approach for infections caused by AmpC-producing organisms [10,11].

Many clinicians avoid the utilization of 3GC due to the potential risk of inducing AmpC beta-lactamases [12]. Carbapenems may be considered the current drug of choice; however, alternatives such as cefepime may also be efficacious [12]. Cefepime demonstrates some stability in the presence of AmpC enzymes and is able to achieve high periplasmic concentrations owing to its zwitterionic structure [2]. Emerging concerns regarding overutilization of carbapenems and antipseudomonal beta-lactams underscore the need to better define the utility of 3GC, such as ceftriaxone, in the management of AmpC-producing organisms [13,14,15]. The purpose of this study is to compare the risk of overall treatment failure (OTF) with definitive 3GC therapy compared to other antibiotic therapy in patients with bloodstream infections (BSIs) due to chromosomally-mediated AmpC-producing Enterobacterales (CAE).

## 2. Results

### 2.1. Participants

**Figure 1:**
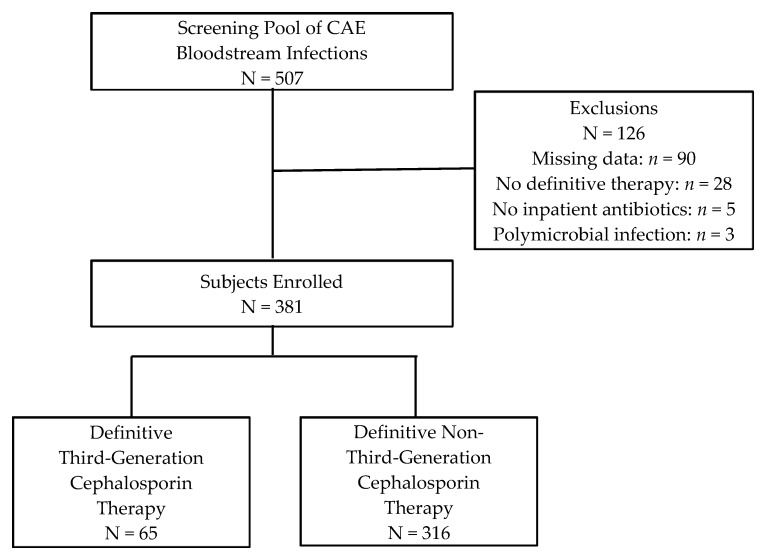
Flow diagram of subject enrollment.

Overall, 507 patients with a BSI secondary to *Enterobacter*, *Serratia,* or *Citrobacter* spp. from 1 January 2006 to 1 September 2014 were screened for study inclusion. Of these, 126 patients were excluded, primarily due to missing data (Figure 1). Of 381 patients enrolled in the study, 65 patients received definitive 3GC therapy, and 316 patients received other antimicrobial agents for definitive therapy. The study population was generally similar between 3GC vs. non-3GC groups (Table 1). Median age was 60 years, and approximately half of the patients were African American. Urinary tract or central venous catheter (CVC) were the most common sources of BSIs, jointly representing 41.0% (156) of cases. Charlson comorbidity index score was comparable between 3GC and non-3GC groups (median 3 vs. 4, respectively, *p* = 0.98). The 3GC group had significantly more patients with renal dysfunction (chronic kidney disease 33.9% vs. 21.2%, *p* = 0.03; median creatinine clearance 45 mL/min vs. 56 mL/min, *p* = 0.04). Conversely, the 3GC group had significantly fewer males (49.2% vs. 63.9%, *p* = 0.03), fewer respiratory sources of BSIs (4.6% vs. 13.3%, *p* = 0.049), and lower severity of illness (ICU admissions 29.2% vs. 45.6%, *p* = 0.02; median Pitt Bacteremia score 2 vs. 3, *p* = 0.04).

*Enterobacter* spp. were the most common bloodstream isolates (62.5%), followed by *Serratia* spp. (27.3%) and *Citrobacter* spp. (10.2%). Table 2 describes the number of isolates by treatment group. The overall distribution of CAE organisms between groups was significantly different (*p* = 0.03), with a numerically higher proportion of *Enterobacter* spp. in the non-3GC group and *Serratia* or *Citrobacter* spp. in the 3GC group.

Overall antibiotic usage was evaluated (Table 3); the mean number of antibiotic agents used was slightly higher in the 3GC group vs. non-3GC group (3.5 vs. 3.0 antibiotic agents, respectively, *p* = 0.005). Inpatient treatment was continued for a median of 10.3 days in the 3GC group, compared to 8.8 days in the non-3GC group (*p* = 0.58). Definitive therapy with 3GCs (primarily ceftriaxone) in the 3GC group composed a median of 70% of the inpatient definitive treatment duration. Thus, for every 7 days of inpatient definitive therapy, a 3GC was used for approximately 5 of those days in most cases (compared to 0 days in the non-3GC group). Among the 3GC group, 64% of ceftriaxone was ordered at a dose of 1 g IV and 36% of ceftriaxone was ordered as 2 g IV, with either dose typically given once daily. Patients in the non-3GC group most commonly received fluoroquinolones, carbapenems, or extended-spectrum penicillins for definitive therapy. Doses varied, but the vast majority were in accordance with package insert dosing recommendations for each respective agent. Baseline non-susceptibility rates to 3GC were 7.7% and 17.7% in the 3GC and non-3GC groups, respectively (*p* = 0.045). Approximately half of the patients were discharged from the hospital on antibiotic therapy (52.3% vs. 51.1%, *p* = 0.86).

### 2.2. Clinical Outcomes

The crude estimate of the primary endpoint of OTF was comparable between the definitive 3GC and non-3GC therapy groups (33.9% vs. 29.8%, respectively, *p* = 0.51). The components of the composite endpoint between definitive 3GC and non-3GC therapy groups, i.e., in-hospital mortality, hospital readmission within 30 days, and reinfection within 90 days, were also comparable (Table 4). The median length of hospital stay was 12.7 vs. 14.2 days in the definitive 3GC and non-3GC therapy groups, respectively (*p* = 0.16). 

**Figure 2:**
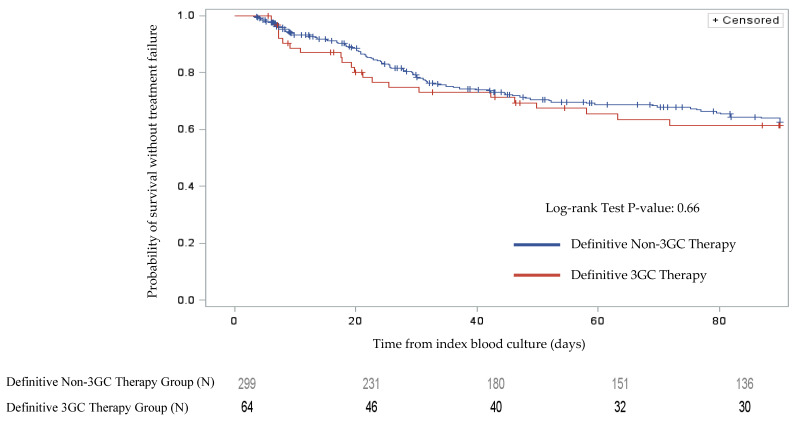
Kaplan–Meier curves for overall treatment failure by therapy group. The red line represents patients on definitive third-generation cephalosporins, and the blue line represents patients on other definitive antibiotics.

The univariable Cox proportional hazards regression model estimated comparable odds of OTF with the definitive 3GC therapy compared to definitive non-3GC therapy (odds ratio (OR) 1.11, 95% confidence interval (CI) 0.70–1.76, *p* = 0.66). Kaplan–Meier curves for OTF by therapy group were comparable (Figure 2, log rank *p* = 0.66). The final multivariable Cox proportional hazards regression model included definitive 3GC therapy and additional covariates such as adequate empiric therapy, male sex, creatinine clearance, source of infection, and site of acquisition (Table 5). This analysis suggested definitive 3GC therapy was not independently associated with OTF (adjusted OR 0.93, 95% CI 0.51–1.72, *p* = 0.83) when compared to definitive non-3GC therapy. There was a trend of higher OTF in patients with malignancy, but it did not achieve statistical significance. No other significant predictors of OTF were identified. Of the 37 patients with an evaluable repeat positive blood culture (10% of the total study population), two isolates demonstrated the emergence of antimicrobial resistance, both in the non-3GC antibiotic group. As a brief post hoc sensitivity analysis, the crude estimate of OTF was evaluated among the subgroup of patients with *Enterobacter* spp. and was comparable between the definitive 3GC and non-3GC therapy groups (24.2% (8/33) vs. 29.2% (60/205), *p* = 0.55).

## 3. Discussion

Reports of higher treatment failure rates in the literature associated with 3GC therapy for *Enterobacter* spp. BSIs have led many clinicians to advise against the use of third-generation cephalosporins in infections with these pathogens, regardless of in vitro susceptibility [12]. This may encourage the utilization of carbapenems for definitive treatment of BSIs due to *Enterobacter* spp. and other CAE. Although the effectiveness of some carbapenem-sparing antimicrobial options has been recently demonstrated, all of the agents utilized were antipseudomonal beta-lactams, such as cefepime or piperacillin–tazobactam [10,16]. This has limited the ability to de-escalate patients receiving antipseudomonal beta-lactams in even the most robust antimicrobial stewardship settings [17]. In an era of increasing antimicrobial resistance and reporting of antimicrobial utilization and resistance to the National Healthcare Safety Network, antimicrobial stewardship programs have been searching for safe strategies to reduce unnecessary utilization of carbapenems and antipseudomonal beta-lactams. This study sought to qualify the consequence of using 3GCs as definitive therapy for BSIs due to organisms frequently associated with the production of AmpC beta-lactamases. The majority of patients in the current study received ceftriaxone, a beta-lactam without antipseudomonal coverage. In contrast to previous literature, our study did not reveal a significant difference in overall treatment failure when 3GCs were used as definitive therapy for BSIs with these pathogens. Overall treatment failure was similar in both treatment arms regardless of antimicrobial selection and with an apparent low risk of emergence of resistance in this population. However, since, by design, all of the patients on definitive 3GC therapy in the current study received intravenous 3GCs (predominantly ceftriaxone) for at least the inpatient portion of therapy, these results should not be generalized to oral 3GCs. Previous studies demonstrated oral beta-lactams were associated with the highest treatment failure rates among all oral options in patients with gram-negative BSIs [18,19]. In addition, these data may not be generalizable to CAE isolates that exhibit higher cefepime minimal inhibitory concentrations (MICs) of 4 or 8 mcg/mL. Such isolates would be interpreted as being “susceptible dose-dependent” to cefepime and recent data has shown that definitive cefepime therapy against such isolates was associated with increased mortality compared to definitive carbapenem therapy [20]. It is unclear if such an association extends to 3GC therapy, yet caution would be prudent until further research can be performed in this subset of isolates.

Additional important considerations are the source of BSIs and the initial bacterial burden [11]. Primary sources in this cohort were the urinary system and central venous catheters (41.0% overall, 20.5% each). Urinary tract and central venous catheter infections are associated with a lower risk of mortality than other sources of Gram-negative BSIs, as demonstrated in previous studies [21,22]. Relatively few patients in the current study had other sources of BSIs compared to prior studies by Chow and colleagues (38.8% abdominal) or Kaye and colleagues (36.5% wounds, 24.9% respiratory) [5,6]. This difference may have been due to the current cohort representing not just university-affiliated and tertiary care referral medical centers, but also community hospitals. Thus, the equivocal findings of definitive 3GC therapy in the current study appear promising, particularly for uncomplicated cases of CAE BSI. This should be a significant help to antimicrobial stewardship programs and others attempting to spare widespread use of carbapenems and antipseudomonal beta-lactams. Additional research is indeed warranted in patients with CAE BSI due to high mortality risk and/or uncontrolled sources of CAE BSI, e.g., intraabdominal infections, meningitis, osteomyelitis, pneumonia, retained prosthetic material. Carbapenems and other AmpC stable beta-lactams, such as cefepime, may continue to be preferred in that setting until sufficient data in such patients are available. A recent cohort study supports this caution, noting risk factors for relapsed or persistent BSIs with *Enterobacter* spp. were immunosuppression or a line-associated source of BSIs [16]. The authors hypothesized delayed line removal, i.e., an uncontrolled source, was the primary driver of the latter association. The current study was designed to evaluate definitive antimicrobial therapy received after 72 h of collection of index blood culture. Patients who did not survive the first 3 days of BSIs were not included in the study. This likely limited the study’s ability to detect previously established risk factors for mortality such as malignancy, liver cirrhosis, source of BSI, and acute severity of illness [21,22].

The multicenter design represents the primary strength of this study. The large number of patients with CAE BSI from 18 medical centers served to increase statistical power and generalizability. These medical centers in the Southeastern Research Group Endeavor (SERGE-45) network included a wider array of small community hospitals, which are typically underrepresented in the medical literature. This makes the data more generalizable to a broader number of institutions across the United States. However, the low incidence rate of BSIs secondary to *Enterobacter* species (3.3 per 100,000 person-years) and other CAE in population-based settings implied a relatively long study period to accrue a sufficient number of patients [23]. Only 381 patients were enrolled, and only 65 patients (17%) received 3GCs for definitive therapy over an 8-year period. This highlights the difficulty of conducting this type of research among sites that include community-based medical centers. In addition, prescribers were less likely to use 3GCs for definitive therapy, particularly in critically-ill patients or high mortality-risk infections, such as respiratory source and *Enterobacter cloacae* BSI. This was likely influenced by healthcare providers’ awareness of prior literature and concern regarding the use of 3GCs, especially in patients at high risk for treatment failure. Moreover, differences in susceptibility reporting by each institution’s microbiology laboratory could have also impacted the choice of definitive therapy as some hospitals may not report susceptibilities to 3GC due to the same concerns. Other limitations include those inherent to retrospective, observation data collection, such as missing data and the large variety of antibiotic agents used in each patient case. Outcome assessments were limited by a lack of source control assessment in the retrospective chart review. However, almost 40% were not amenable to source control (e.g., respiratory, other, unknown source). A randomized clinical trial comparing ceftriaxone to a carbapenem for definitive therapy of CAE BSI represents the ultimate design in order to eliminate confounding by indication, among other limitations of observational cohort studies. However, the availability of very effective and highly bioavailable oral options for treatment will make enrollment in such a clinical trial unattractive to the majority of patients with BSI due to fluoroquinolone-susceptible organisms [18,19,24]. In addition, pharmacokinetic differences between antimicrobial agents utilized were not assessed and, thus, meaningful conclusions about dosing strategies in the 3GC group could not be made. Finally, potential AmpC production was defined based on phenotypic rather than molecular testing methods.

## 4. Materials and Methods

### 4.1. Study Design and Population

This was a multicenter, retrospective, observational cohort study conducted in hospitalized patients. Study sites were enrolled from within the SERGE-45 network and represented 18 institutions from across the Southeast. The study protocol was approved by the local institutional review board (IRB) within the Prisma Health System, which served as the coordinating site for the study (IRB identification code Pro00039225, approval date 22 October 2014). Patients with blood cultures that were positive for select CAE organisms (i.e., *Enterobacter*, *Serratia*, or *Citrobacter* spp.) between 1 January 2006 and 1 September 2014 were screened for study inclusion. This included *Enterobacter aerogenes* (now known as *Klebsiella aerogenes*). The earliest positive blood culture was deemed the index blood culture. Additional inclusion criteria were patient age ≥18 years and initiation of antibiotic therapy following positive blood culture. Exclusion criteria included polymicrobial BSI based on the index blood culture (exception: coagulase-negative *Staphylococcus* spp. or diphtheroids deemed a contaminant, as suggested by the Infectious Diseases Society of America guidelines and the treatment team), death prior to speciation or within 72 h of index blood culture, transfer from an outside hospital with previous blood culture positive for target CAE organisms, pregnancy, incarceration, previous study enrollment for a prior episode of BSI, or missing data that would preclude the appropriate determination of a study group or primary endpoint [25].

### 4.2. Study Definitions

CAE BSI was defined as the growth of *Enterobacter* spp., *Serratia* spp., or *Citrobacter* spp. in a blood culture. Potential AmpC beta-lactamase production was based on phenotypic testing of bloodstream isolates as previously defined [24]. Empiric therapy was defined as any antibiotic use within the first 72 h from the date/time of index blood culture collection, and definitive therapy was defined as any antibiotic use thereafter (i.e., greater than 72 h after index blood culture). Definitive 3GC therapy was specifically defined as any 3GC use (e.g., ceftriaxone, ceftazidime, cefotaxime) during the time period greater than 72 h after index blood culture collection, whereas definitive non-3GC therapy was defined as any antibiotic therapy which excluded 3GCs during the same period. Source of BSI was based on physician documentation in the medical record. Source of infection other than urinary tract or CVC was characterized as one variable based on prior evidence identifying this as a risk factor for mortality in Gram-negative BSIs based on its association with a higher burden of infection [21,22].

Overall treatment failure was the primary composite endpoint consisting of in-hospital mortality, hospital readmission within 30 days of hospital discharge, or recurrent infection with the same organism within 90 days of hospital discharge (source of the infection may have differed). Adequate therapy (modified from Cain et al.) [26] was determined as meeting each of the following criteria: (1) antibiotic agent was administered intravenously, with the exception of oral fluoroquinolones which were considered appropriate in hemodynamically stable patients due to high oral bioavailability, and (2) the bloodstream isolate demonstrated in vitro susceptibility to at least one administered antibiotic agent based on Clinical and Laboratory Standards Institute (CLSI) interpretive criteria. Emergence of resistance was defined as an isolate that was initially susceptible to a specific antibiotic agent, then became non-susceptible (i.e., intermediate or resistant) to the agent upon repeat culture of the index organism.

### 4.3. Study Endpoints

The primary endpoint was overall treatment failure. Definitive 3GC therapy was compared to definitive non-3GC therapy with an analysis of additional potential risk factors for OTF. Secondary endpoints included the individual components of the composite endpoint of OTF as well as 30-day mortality, hospital length of stay, and the emergence of antimicrobial resistance.

### 4.4. Microbiology Procedures & Data Management

Identification of bacterial isolates and antimicrobial susceptibility testing were performed at the local microbiology laboratory for each participating medical center in accordance with CLSI guidelines.

Pharmacy and/or microbiology software systems at respective institutions were utilized to retrospectively identify positive bloodstream cultures with *Enterobacter, Serratia*, and/or *Citrobacter* spp. within the defined study period. Local electronic medical records at each study site were used to collect patient data in a retrospective fashion for both study groups. REDCap™, an online data management tool, was used for standardized data collection [27].

### 4.5. Statistical Analysis

Baseline categorical variables were analyzed via the chi-square test or Fisher’s exact test, as appropriate. Baseline continuous variables were analyzed using Mann–Whitney U or Student’s *t*-test, as appropriate. OTF was analyzed via Kaplan–Meier survival curves by therapy group. Univariable and multivariable Cox proportional hazards regression was performed to evaluate the effect of definitive 3GC therapy on OTF, while controlling for potential confounders. Patients who did not experience OTF during the study follow-up period were censored upon last documented interaction with the healthcare system or at the end of the 90-day follow up period. Any covariate with a *p*-value ≤0.2 was included in the final regression model. Backward selection methods, along with findings from the univariable analyses and published data determined a priori, were used to determine the most parsimonious model with the best fit of the data. Given the particularly notable risk of emergence of resistance with *Enterobacter* spp. compared to other CAE [5,6,11], a post hoc sensitivity analysis on the subgroup of patients with *Enterobacter* spp. BSI was performed for the primary endpoint of OTF. Statistical analysis was performed using SAS, version 9.4 (SAS Institute Inc., Cary, NC, USA).

For the primary endpoint of OTF, it was estimated the study would have to enroll 50 patients in the definitive 3GC therapy group and 250 patients in the definitive non-3GC therapy group (N = 300 patients total with an estimated enrollment ratio of 1:5). This would provide 80% power to detect a 20% increase in the OTF rate in the definitive 3GC group vs. the definitive non-3GC therapy group, assuming a baseline OTF rate of 25% in the definitive non-3GC therapy group and a two-sided alpha level of 0.05. This delta was based on prior data demonstrating the rate of emergence of resistance was 19% with the use of broad-generation cephalosporins [6].

A STROBE statement outlining a completed checklist of items included in this report as a cohort study is provided in the supplementary materials [28].

## 5. Conclusions

Our study demonstrates similar risk of overall treatment failure with definitive 3GC therapy compared to other antimicrobial therapies for BSI due to select organisms with the potential to produce AmpC (i.e., *Enterobacter*, *Serratia*, and *Citrobacter* spp.). These findings should prompt further investigation into the role of third-generation cephalosporins in the treatment of infections with these organisms. These may serve as important options to help spare widespread use of carbapenems and other antipseudomonal beta-lactams.

## Figures and Tables

**Table 1:** Baseline characteristics of patients on definitive therapy with third-generation cephalosporin (3GC) versus other antimicrobial agents.

Characteristic	Definitive 3GC Therapy ^a^ N = 65	Definitive Non-3GC Therapy ^a^ N = 316	*p*-Value
Age, y (median (IQR))	59 (46.5–68.0)	60 (47.8–70.0)	0.989
Male	32 (49.2)	202 (63.9)	0.027
Weight, kg (median (IQR))	80 (71.0–102.3)	85 (70.0–103.0)	0.884
Race/Ethnicity			0.308
Black	33 (50.8)	130 (41.4)	
White	29 (44.6)	175 (55.4)	
Other/Unknown	3 (4.6)	11 (3.5)	
Intensive Care Unit Admission	19 (29.2)	144 (45.6)	0.016
CrCl, mL/min (median (IQR))	45 (21.9–85.4)	56 (28.7–86.7)	0.036
Penicillin/Cephalosporin Allergy	8 (12.3)	41 (13.0)	0.884
Charlson Comorbidity Index (median (IQR))	3 (2.0–6.0)	4 (2.0–6.0)	0.984
Source of Infection			
Central Venous Catheter	17 (26.2)	61 (19.3)	0.213
Urinary Tract	15 (23.1)	63 (19.9)	0.568
Skin- or Skin Structure-Related	9 (13.9)	35 (11.1)	0.525
Intra-Abdominal	6 (9.2)	34 (10.8)	0.714
Respiratory	3 (4.6)	42 (13.3)	0.049
Other	6 (9.2)	16 (5.1)	0.237
Unknown Source	9 (13.9)	76 (24.1)	0.072
Site of Acquisition			0.275
Hospital-Acquired	27 (42.2)	160 (50.6)	
Healthcare-Associated	21 (32.8)	102 (32.3)	
Community-Acquired	16 (25.0)	54 (17.1)	
Pitt Bacteremia Score (median (IQR))	2 (1.0–3.0)	3 (1.0–4.0)	0.043
Adequate Empiric Therapy	60 (92.3)	291 (92.1)	0.952

Abbreviations: 3GC, third-generation cephalosporin; IQR, interquartile range; CrCl, creatinine clearance. ^a^ Data reported as *n* (%) unless otherwise noted.

**Table 2:** Microbiology of bloodstream infections by therapy group.

Organism	Total ^a^ N = 381	Definitive 3GC Therapy ^a,b^ N = 65	Definitive Non-3GC Therapy ^a,b^ N = 316
*Enterobacter* spp.	238 (62.5)	33 (50.7)	205 (64.8)
*E. cloacae*	142 (37.3)	19 (29.2)	123 (38.9)
*E. aerogenes*	82 (21.5)	14 (21.5)	68 (21.5)
Other *Enterobacter* spp.	14 (3.7)	0 (0)	14 (4.4)
*Serratia* spp.	104 (27.3)	21 (32.3)	83 (26.2)
*S. marcescens*	100 (26.2)	19 (29.2)	81 (25.6)
Other *Serratia* spp.	4 (1.0)	2 (3.1)	2 (0.6)
*Citrobacter* spp.	39 (10.2)	11 (16.9)	28 (8.8)
*C. freundii*	21 (5.5)	8 (12.3)	13 (4.1)
Other *Citrobacter* spp.	18 (4.7)	3 (4.6)	15 (4.7)

^a^ Data reported as *n* (%). ^b^ *p* < 0.05 when evaluating the distribution of *Enterobacter*, *Serratia*, and *Citrobacter* spp between both groups.

**Table 3:** Antibiotic utilization by therapy group.

Variable	Definitive 3GC Therapy ^a^ N = 65	Definitive Non-3GC Therapy ^a^ N = 316	*p*-Value
Number of Inpatient Antibiotics Used (mean (SD))	3.5 (1.6)	3.0 (1.4)	0.005
Antibiotic Class			
Extended-Spectrum Penicillins ^b^	30 (46.2)	187 (59.2)	0.054
Third-Generation Cephalosporins	65 (100)	31 (9.8) ^c^	<0.001
Fourth-Generation Cephalosporins	12 (18.5)	83 (26.3)	0.185
Carbapenems	16 (24.6)	115 (36.4)	0.069
Fluoroquinolones	25 (38.5)	158 (50.0)	0.090
Aminoglycosides	12 (18.5)	54 (17.1)	0.790
Other Antibiotics	40 (61.5)	182 (57.6)	0.580
Inpatient Gram-Negative Antibiotic Duration, days (median (IQR))	10.3 (5.7–15.6)	8.8 (5.0–13.9)	0.578
Empiric Duration	2.8 (2.3–3.0)	2.7 (2.2–3.0)	0.470
Definitive Duration	7.4 (3.5–12.9)	5.8 (2.4–11.0)	0.620
Inpatient 3GC Duration, days (median (IQR))	4.4 (2.8–7.4)	0.5 (0.5–1.6)	<0.001
Empiric 3GC Duration	2.5 (0.9–3.0)	0.5 (0.5–1.6)	0.002
Definitive 3GC Duration	3.3 (1.4–6.5)	--	--
Proportion of Time 3GC Used for Definitive Inpatient Therapy (median (IQR))	0.7 (0.3–1.0)	--	--
Adequate Definitive Therapy ^d^			0.487
Yes	63 (98.4)	281 (95.3)	
No	1 (1.6)	14 (4.7)	
Number of Patients Discharged on Antibiotics	34 (52.3)	161 (51.1)	0.861

Abbreviations: 3GC, third-generation cephalosporin; IQR, interquartile range; SD, standard deviation. ^a^ Data reported as *n* (%), unless otherwise noted. ^b^ Including extended-spectrum penicillins and beta-lactamase inhibitor combinations. ^c^ Empiric 3GC use only (no definitive 3GC use) ^d^ Due to missing data, N = 64 in definitive 3GC therapy group and N = 295 in definitive non-3GC therapy group

**Table 4:** Clinical outcomes by therapy group.

Outcome	Definitive 3GC Therapy ^a^ N = 65	Definitive Non-3GC Therapy ^a^ N = 316	*p*-Value
Overall Treatment Failure	22 (33.8)	94 (29.7)	0.513
In-Hospital Mortality	4 (6.2)	33 (10.4)	0.302
Hospital Readmission within 30 Days	15 (23.1)	53 (17.0)	0.245
Reinfection within 90 Days	6 (9.2)	16 (5.1)	0.232
30-Day Mortality	4 (6.2)	27 (8.5)	0.521
Length of Hospital Stay, days (median (IQR))	12.7 (7.7–32.6)	14.2 (6.7–34.9)	0.158

Abbreviations: 3GC, third-generation cephalosporin; IQR, interquartile range. ^a^ Data reported as *n* (%), unless otherwise noted.

**Table 5:** Multivariable Cox proportional hazards regression model for overall treatment failure.

Variable	Adjusted HR	95% CI	*p*-Value
Definitive 3GC Therapy ^a^	0.93	0.51–1.72	0.825
Adequate Empiric Therapy	0.40	0.12–1.32	0.132
Male Sex	0.67	0.42–1.08	0.097
CrCl, per mL/min increase	1.00	0.99–1.08	0.223
Source of Infection other than Urinary Tract or CVC	1.04	0.65–1.67	0.858
Site of Acquisition			
Community-Acquired	Ref.	-	-
Healthcare-Associated	0.85	0.46–1.57	0.596
Hospital-Acquired	1.13	0.59–2.17	0.714
Malignancy	1.71	0.98–2.99	0.059
Liver Cirrhosis	1.88	0.80–4.46	0.151
Pitt Bacteremia Score, per point increase	1.04	0.93–1.17	0.488
Penicillin/Cephalosporin Allergy	1.30	0.80–2.13	0.292

Abbreviations: HR, hazard ratio; CI, confidence interval; 3GC, third-generation cephalosporin; CrCl, creatinine clearance; CVC, central venous catheter. ^a^ Definitive non-3GC therapy as the referent group.

## Supplementary Materials

The following is available online at https://www.mdpi.com/2079-6382/9/5/254/s1, Table S1: STROBE Statement—Completed checklist of items that should be included in reports of cohort studies.
